# Adaptation of SIVmac to baboon primary cells results in complete absence of *in vivo* baboon infectivity

**DOI:** 10.3389/fcimb.2024.1408245

**Published:** 2024-06-28

**Authors:** Veronica Obregon-Perko, Amanda Mannino, Jason T. Ladner, Vida Hodara, Diako Ebrahimi, Laura Parodi, Jessica Callery, Gustavo Palacios, Luis D. Giavedoni

**Affiliations:** ^1^ Texas Biomedical Research Institute, San Antonio, TX, United States; ^2^ The Pathogen and Microbiome Institute, Northern Arizona University, Flagstaff, AZ, United States; ^3^ Department of Biology, Trinity University, San Antonio, TX, United States; ^4^ Icahn School of Medicine at Mount Sinai, New York, NY, United States

**Keywords:** viral adaptive changes and evolution, simian immunodeficiency virus (SIV), baboon, innate immunity, chronic infection

## Abstract

While simian immunodeficiency virus (SIV) infection is non-pathogenic in naturally infected African nonhuman primate hosts, experimental or accidental infection in rhesus macaques often leads to AIDS. Baboons, widely distributed throughout Africa, do not naturally harbor SIV, and experimental infection of baboons with SIVmac results in transient low-level viral replication. Elucidation of mechanisms of natural immunity in baboons could uncover new targets of antiviral intervention. We tested the hypothesis that an SIVmac adapted to replicate in baboon primary cells will gain the capacity to establish chronic infections *in vivo*. Here, we generated SIVmac variants in baboon cells through serial passage in PBMC from different donors (SIVbn-PBMC s1), in PBMC from the same donors (SIVbn-PBMC s2), or in isolated CD4 cells from the same donors used for series 2 (SIVbn-CD4). While SIVbn-PBMC s1 and SIVbn-CD4 demonstrated increased replication capacity, SIVbn-PBMC s2 did not. Pharmacological blockade of CCR5 revealed SIVbn-PBMC s1 could more efficiently use available CCR5 than SIVmac, a trait we hypothesize arose to circumvent receptor occupation by chemokines. Sequencing analysis showed that all three viruses accumulated different types of mutations, and that more non-synonymous mutations became fixed in SIVbn-PBMC s1 than SIVbn-PBMC s2 and SIVbn-CD4, supporting the notion of stronger fitness pressure in PBMC from different genetic backgrounds. Testing the individual contribution of several newly fixed SIV mutations suggested that is the additive effect of these mutations in SIVbn-PBMC s1 that contributed to its enhanced fitness, as recombinant single mutant viruses showed no difference in replication capacity over the parental SIVmac239 strain. The replicative capacity of SIVbn-PBMC passage 4 (P4) s1 was tested *in vivo* by infecting baboons intravenously with SIVbn-PBMC P4 s1 or SIVmac251. While animals infected with SIVmac251 showed the known pattern of transient low-level viremia, animals infected with SIVbn-PBMC P4 s1 had undetectable viremia or viral DNA in lymphoid tissue. These studies suggest that adaptation of SIV to grow in baboon primary cells results in mutations that confer increased replicative capacity in the artificial environment of cell culture but make the virus unable to avoid the restrictive factors generated by a complex multicellular organism.

## Introduction

Simian immunodeficiency virus (SIV) infection in rhesus macaques (*Macaca mulatta*) of Indian origin is the most extensively used and best characterized NHP model for AIDS. SIVmac251 and SIVmac239 were originally isolated from accidentally-infected rhesus macaques and so are well-adapted for growth in this species (Daniel et al., 1985; Kestler et al., 1990). Experimental SIVmac infection of rhesus macaques leads to high viremia, CD4 T cell depletion, and opportunistic infections. Although the outcome of infection is typically uniform across animals, some genetic correlates of protection and disease progression have been identified, including allelic variations in MHC and TRIM-5α (Yant et al., 2006; Loffredo et al., 2007; Kirmaier et al., 2010; Lim et al., 2010a, b). SIV’s naturally infect over 45 species of African NHP, including African green monkeys (*Chlorocebus* spp.) and sooty mangabeys (*Cercocebus atys*) (VandeWoude and Apetrei, 2006; Klatt et al., 2012). Unlike SIVmac infection in Asian macaques, SIV infection in natural hosts generally does not cause disease, despite active viral replication (Rey-Cuille et al., 1998; Broussard et al., 2001; Pandrea and Apetrei, 2010; Klatt et al., 2012). Viral persistence in the absence of pathology in the natural host is the result of thousands of years of co-evolution with the virus (Worobey et al., 2010). Long-term host-pathogen interactions are also reflected by the evolution of species-specific SIV strains, each adapted to optimally circumvent restriction factors in their respective host species (VandeWoude and Apetrei, 2006; Kirmaier et al., 2010; Krupp et al., 2013). Differences in disease outcome between natural and non-natural SIV hosts cannot be explained by immune control of viral replication, as natural hosts have viral loads similar to those of humans and macaques during pathogenic infection (Rey-Cuille et al., 1998; Broussard et al., 2001; Silvestri et al., 2003; Dunham et al., 2006). Instead, immunomodulation and altered virus cell type tropism are believed to be the major mechanisms by which pathology is limited (Chahroudi et al., 2012; Jasinska et al., 2022).

Baboons (*Papio hamadryas* sp.) are abundant and widespread in Africa but are not known to be endemically infected with SIV. This is unexpected when considering over 90% of African NHP species tested harbor a species-specific SIV (Peeters et al., 2014). The geographical distribution of baboon subspecies vastly overlaps with that of natural NHP hosts, especially African green monkeys (Zinner et al., 2009; Chahroudi et al., 2012). Furthermore, a significant portion of a baboon’s diet is comprised of African green monkeys, a species where SIV prevalence is roughly 50% (Hausfater, 1976; Ma et al., 2013). Field studies have provided some serological evidence of cross-species transmission between baboons and African green monkeys (Kodama et al., 1989). Thus, despite baboon exposure to SIV through co-habitation and predation, circulating viruses have yet to be recovered from these animals.

Studies on HIV/SIV tropism in baboons have yielded different results depending on the strain of virus. *In vitro* infections with SIVmac demonstrated baboon lymphocytes are susceptible to infection, but virus growth was less efficient than in rhesus macaque lymphocytes (Kannagi et al., 1985; Benveniste et al., 1986). In support of this finding, Cranage et al. showed baboons can support persistent SIVmac infection *in vivo*; however, baboons did not progress to disease or develop any tissue pathology at the microscopic level (Cranage et al., 1992). Baboons challenged with an SIV strain from pig-tailed macaques (SIVMne) showed no clinical signs of disease, had undetectable virus in circulation and tissues, and showed no seroconversion one year post-inoculation (Benveniste et al., 1988). Baboons have also been evaluated for their ability to support infection with simian-human immunodeficiency viruses (SHIVs) made with a SIVmac239 backbone (Allan et al., 1995), however these SHIVs expressed Env from CXCR4-or dual-tropic HIV-1 strains, distinct from the CCR5-tropic nature of SIVs. The infectivity of various HIV-2 isolates, which are genetically similar to SIVsmm and SIVmac, has also been interrogated in baboons; only one isolate, HIV-2_UC2_, consistently induced viremia in baboons (Letvin et al., 1987; Barnett et al., 1994). However, like previously explored SHIVs, HIV-2_UC2_ is a dual-tropic strain and, as such, findings in this model may not be relevant in infection with naturally occurring CCR5-tropic SIVs.

We and others have demonstrated SIVmac replication is restricted in baboon PBMC relative to rhesus macaque PBMC (Kannagi et al., 1985; Obregon-Perko et al., 2018). However, baboon and rhesus macaque isolated CD4 cells are equally permissive to infection. We determined that restriction of SIVmac in baboon PBMC is unlikely to be due to intrinsic intracellular restriction factors (e.g. TRIM-5α), which typically impose barriers to cross-species transmission of SIVs (Kirmaier et al., 2010; Krupp et al., 2013), but instead to innate mechanisms mediated by other lymphocyte types, primarily CD8 T and NK cells. One such mechanism involves elevated production of CCR5-binding chemokines that impede viral docking to CCR5, a co-receptor used by HIV and SIV for entry into CD4 cells (Cocchi et al., 1995). Although we have shown this process contributes to control of viral replication, baboon resistance to SIV infection and/or disease is likely mediated by multiple independent mechanisms.

Immune pressures exerted by the host can drive viral evolution during the course of infection. This molecular arms race is revealed at the amino acid level, as host and viral proteins accumulate changes better suited to defeat their opponent. Viruses have the advantage of rapid evolution due to their short generation time and, in the case of RNA viruses, high mutation rates. Indeed, a previous study reported an HIV-2 strain with increased replication kinetics and virulence after serial passage in baboons (Locher et al., 2003a). However, follow-up studies were not performed to identify changes to the viral genome that could be attributed to adaptation and the HIV-2 strain used in this study was dual-tropic. Interrogating changes to the SIV genome during infection in baboon cells could uncover viral genes under strong selective pressure from baboon antiviral mechanisms. Toward this end, we aimed to generate a baboon-adapted CCR5-tropic SIV through serial passage of SIVmac in baboon primary cells and to analyze changes to the viral genome through deep-sequencing.

## Materials and methods

### PBMC and CD4 cell infections

Whole blood in EDTA was obtained from SIV seronegative baboons (*Papio hamadryas*) bred in the Southwest National Primate Research Center (SNPRC) at the Texas Biomedical Research Institute (TxBiomed). Animal care and treatments were all in accordance with protocols approved by the TxBiomed Institutional Animal Care and Use Committee (IACUC). All animals were confirmed serologically negative for simian T-lymphotropic virus (STLV) and SIV antibodies by Luminex assay (Obregon-Perko et al., 2018). Peripheral blood mononuclear cells (PBMC) were isolated by gradient centrifugation using Lymphocyte Separation Medium (Cellgro, Corning). CD4 cells were sorted from freshly isolated PBMC by positive selection using magnetic beads coated with anti-CD4 (clone L200) antibodies, as per the manufacturer’s instructions (IMag™ Human CD4 T Lymphocyte Enrichment Set-DM, BD Biosciences). Purity of the positive fraction was assessed by flow cytometry using a clone of anti-CD4 antibody that differed from that used for sorting (CD4-APC, clone 13B8.2, Beckman-Coulter).

**Figure 1:**
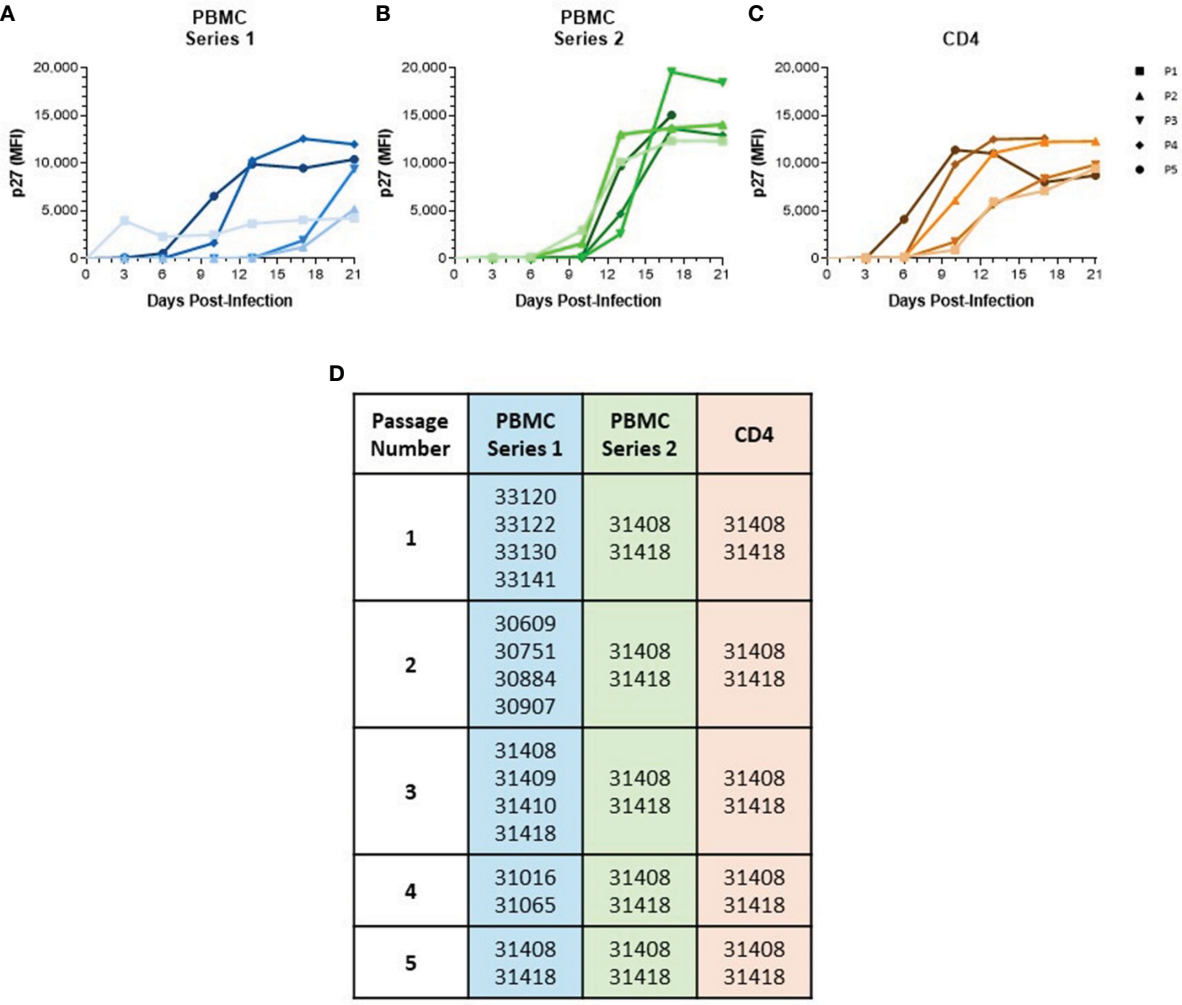
Serial passage of SIVmac in baboon PBMC and isolated CD4 Cells. **(A)** PBMC were isolated from different baboon donors for each viral passage. **(B)** PBMC and **(C)** CD4 cells were isolated from the same two baboon donors for each viral passage. All cells were cultured for 48 hr in RPMI-10 containing PHA then infected with SIVmac251 at a M.O.I. of 100 g.e./cell to generate passage 1. Generated virus was then used to continue passage series at same M.O.I. Viral loads were determined by measuring p27 median fluorescence intensity in the supernatant by Luminex assay. **(D)** List of baboon identifiers used for different viral passages.

Cells were cultured in complete RPMI-10 containing 5 μg/mL PHA for 48 hr prior to being infected by Magnetofection™ (OZ Biosciences), as described previously (Obregon-Perko et al., 2018). For the first passage, 4 x 10^6^ PBMC or CD4 cells, pooled from 2–4 donors, were infected with SIVmac251 (referred to as Passage 0 or P0) at a multiplicity of infection (M.O.I.) of 100 g.e./cell. Infections were maintained for about 21 d in RPMI 1640 (Corning) with 10% fetal bovine serum (RPMI-10) supplemented with 50 U/mL IL-2 (NIH AIDS Reagent Program). Supernatant harvested at time points with the highest viral loads were pooled to generate a viral stock referred to as SIV baboon passage 1 (SIVbn P1). Viruses passaged in PBMC were denoted as SIVbn-PBMC and those passaged in CD4 cells as SIVbn-CD4. Viral titer of SIVbn P1 was quantified by real-time RT-PCR. Freshly isolated PBMC or CD4 cells were then infected with SIVbn P1 at a M.O.I. of 100 g.e./cell to continue the passage series. Serial passage was performed five times, with the final passage generating SIVbn P5. In the first PBMC passage series, series 1, different baboon donors were used for each passage. In PBMC series 2 and CD4 passages, the same two baboon donors were used for each passage. Animals used for each series and passage are listed in **Figure 1D**. For donor-matched infections, PBMC and CD4 cells were isolated from the same two baboon donor. After 48 hr in culture, 1 x 10^6^ cells were contemporaneously infected with SIVmac251, SIVbn-PBMC, or SIVbn-CD4 at a M.O.I. of 100 g.e./cell. All cell cultures were maintained at 2 x 10^6^ cells/mL in RPMI-10 + IL-2. Half of the medium was replenished twice a week. Infections were monitored by measuring the levels of SIV p27 in the supernatant by Luminex assay (Obregon-Perko et al., 2018).

### Virus genome deep sequencing

Samples were lysed in TRIzol LS at a 1:3 ratio and RNA was extracted with either the Direct-zol RNA MiniPrep (ZYMO) or Phasemaker tubes (Invitrogen) with the PureLink RNA mini Kit (ThermoFisher) following manufacturer’s protocols. A subset of the samples was DNase-treated using the PureLink DNAse set (ThermoFisher). Samples with low viral loads were enriched for viral RNA by depleting host rRNA with DNA probes complementary to human rRNA. RNAseq libraries were prepared using the library prep portion of the Truseq RNA access Library Prep (now TruSeq RNA Exome) kit (Illumina) with the following modifications: fragmentation time varied from 30 sec - 4 min at 94°C depending on the integrity of the RNA input; SuperScript IV RT was used (ThermoFisher); custom dual indexes were used; a total of 20 cycles were done for PCR Amplification. A final PCR clean-up was done using 0.7X AmpureXP beads to remove index dimers. Libraries were quantified using Tapestation Agilent D1000 ScreenTape assay for nM concentration followed by manual normalization to 2 nM. qPCR was performed on individual libraries with the KAPA Library Quantification Kit Illumina platforms (Kapa Biosystems). Libraries were normalized based on qPCR results and pooled for sequencing on either an Illumina MiSeq or NextSeq platform.

Raw sequencing reads were first filtered based on the quality of the two index reads (average quality ≥20) to help prevent bleed-through between multiplexed samples. Cutadapt v.1.9.dev1 (Martin and Wang, 2011) was then used to remove the random hexamer associated with read one and the Illumina adaptors, and Prinseq-lite v.0.20.3 (Schmieder and Edwards, 2011) was used to filter low-quality reads and bases. SPAdes 3.7.1 (Bankevich et al., 2012) with default settings was used to conduct *de novo* viral genome assemblies for the initial starting material (SIVmac251/P0) and all subsequent cell culture passages (SIVbn P1-P5). For each sample, SIV contigs were identified using BLAST (NCBI), overlapping contigs were joined using Sequencher v5.2.3 (Gene Codes) and when long terminal repeats were joined by the assembler, these were manually separated. Consensus genomes were generated by mapping preprocessed reads to the final *de novo* assemblies using Bowtie2 v.2.0.6 (Langmead and Salzberg, 2012), Samtools v0.1.18 (Li et al., 2009) and custom scripts (https://github.com/jtladner/Scripts/blob/master/reference-based_assembly/consensus_fasta.py). Prior to consensus determination, duplicate reads were removed using Picard (http://broadinstitute.github.io/picard). Only bases with Phred quality score ≥20 were used in consensus calling, and a minimum of 3 × read-depth coverage, in support of the consensus, was required to make a call; positions lacking this depth of coverage were treated as missing (called as ‘N’).

For intrahost single nucleotide variant (iSNV) detection, preprocessed reads from each sample were mapped to a single reference genome, the SIVmac251/P0 *de novo* assembly, using the same approach described previously for consensus generation. iSNVs were identified using freeBayes v1.0 (Garrison et al., 2018) with the “pooled-continuous” population model. For an iSNV to be reported we required that an alternative allele be present at ≥3% frequency and be supported by ≥5 reads in at least one sample within a passage series.

### Analysis of APOBEC-induced mutations

The SIV consensus sequences (described above) were aligned to the parental sequence SIVMac251, also referred to as passage 0 (**Supplementary Table 1**). For each passage series and number, the percentage of mutations were calculated. These data were then organized into a matrix (21 passage conditions x 292 mutated sites) and subjected to principal component analysis, which was performed using Matlab R2019a. We also examined both the types of G>A mutations and their sequence context (GG>AG or GA>AA) to gain insights into potential underlying mutational processes.

### Infection of co-receptor indicator cell lines

Co-receptor tropism was investigated using GHOST (Vödrös and Fenyö, 2005, Virology) indicator cell lines expressing CD4 and high levels of a given co-receptor: CCR5, Bonzo/CXCR6, BOB/GPR15, or CXCR4 (NIH AIDS Reagent Program). These cells stably express a tat-dependent HIV-2 LTR green fluorescent protein (GFP) construct that produces GFP during productive infection. 2.5 x 10^4^ cells were seeded in each well of a 24-well plate in 500 μL propagation medium (high glucose DMEM, 90%; FBS, 10%, 500 μg/ml G418, 100 μg/ml hygromycin, 1 μg/ml puromycin, and 1% pen/strep). After overnight incubation, medium was removed and cells were infected with SIVmac251 or SIVbn-PBMC by Magnetofection™ (ViroMag R/L, OZ Biosciences) at a M.O.I. of 10^4^ g.e./cell. Mock conditions received an equal amount of medium containing ViroMag. Infection volume did not exceed 100 μL. Cells were incubated with viral suspension at 37°C for 2 h, washed twice with warm medium, and then cultured in 500 μL propagation medium. At 48 h.p.i., cells were observed by fluorescence microscopy for GFP expression using an Eclipse Ti confocal microscope (Nikon) and NIS Elements Imaging Software.

### Maraviroc treatment

For receptor blockage tests, isolated baboon CD4 cells were left untreated or treated for 1 h with 1 μM of the CCR5 antagonist maraviroc (NIH AIDS Reagent Program). Cells were then infected with SIVmac251 or SIVbn-PBMC at a M.O.I. of 100 g.e./cell. After 2 h, cells were washed then cultured at 2 x 10^6^ cells/mL in complete RPMI-10 + IL-2 medium. Experimental conditions had media supplemented with 1 μM maraviroc. For dose-response tests, baboon CD4 cells were infected with SIVmac251 or SIVbn-PBMC then cultured in medium alone or medium supplemented with 10-fold serial dilutions of maraviroc ranging from 0.1 nM to 1 μM. All infections were maintained for 10–14 d, with half the media and drug replenished twice a week. Viral loads were measured by p27 quantification with Luminex assay.

### Site directed mutagenesis

Individual SIV mutations were introduced into a plasmid containing the SIVmac239 proviral genome, using the Q5 Site-Directed Mutagenesis Kit (New England Biolabs, Cat No: E0554S) following manufacturer specifications. Briefly, non-overlapping primers containing the desired mutations were designed with NEBaseChanger web tool (New England Biolabs, nebasechanger.neb.com) and were custom manufactured (ThermoFisher). **Supplementary Table S.01** contains information on all the primers utilized for the site-directed mutagenesis procedures. Presence of the desired mutation, in the absence of any unintended mutations, was confirmed by Sanger sequencing (Eurofins). For mutations found in the 5’ half of the SIVmac239 genome, pMA239-T3F OpenNef (a plasmid containing the entire SIVmac239 genome that expresses full-length Nef) and pVP-1 (a plasmid containing the 5’ half of SIVmac239) were digested simultaneously with SphI-HF (New England Biolabs, Cat No: R3182L) and BamHI-HF (New England Biolabs, Cat No: R3136L) in CutSmart Buffer 10X (New England Biolabs, Cat No: B7204S). For mutations found in the 3’ half of the SIVmac239 genome, pMA239 OpenNef and pVP−2 OpenNef (a plasmid containing the 3’ half of SIVmac239) mutants were digested simultaneously with SphI-HF and EcoRI-HF (New England Biolabs, Cat No: R3101L) in CutSmart Buffer 10X. Digestion products were run in an agarose gel for fragment separation, and the desired fragment was gel extracted utilizing the QIAquick Gel Extraction Kit (Qiagen, Cat No: 28704). Fragments were eluted in nuclease-free water and stored at −20°C until the ligation procedure. pMA239-T3F OpenNef [SphI to BamHI] fragments and the corresponding pVP−1 mutants [BamHI to SphI], or pMA239 OpenNef [EcoRI to SphI] fragments and the corresponding pVP−2 OpenNef mutants [SphI to EcoRI], were ligated with T4 DNA Ligase (New England Biolabs, Cat No: M0202M) in the provided T4 DNA Ligase Buffer 10X, or CutSmart Buffer 10X supplemented with ATP, according to manufacturer’s recommended procedure.

Human embryonic kidney cells 293T/17 (HEK293T, ATCC CRL-11268) were maintained in DMEM-5, which consists of Dulbecco’s Modified Eagle’s medium (DMEM) (Corning, Cat No: 10–101-CV) supplemented with 5% fetal bovine serum (FBS), 2 mM GlutaMAX (ThermoFisher, Cat No: 35050061), 25 mM HEPES (Gibco, Cat No: 15–630-080), 1 mM sodium pyruvate (Gibco, Cat No: 11–360-070), and 1X nonessential amino acids (Gibco, Cat No: 11–140-050) at 37°C with 5% CO_2_ incubation. Transfections were performed utilizing TransIT-LT1 Transfection Reagent (Mirus Bio, Cat No: MIR 2300) per manufacturer’s protocol. Cells were incubated with the transfection solution for 48 hours. Pseudotyped viruses were harvested by passing the supernatant through a 0.22 μm filter and then were frozen at −80°C.

### Animals and viral challenge

Olive baboons (*Papio anubis*, n=7), approximately 4 y/o, were obtained from the Southwest National Primate Research Center baboon breeding colony. The animals were fed and housed according to regulations set forth by the Guide for the Care and Use of Laboratory Animals and the Animal Welfare Act, and the animal experiments were approved by the TxBiomed IACUC. Serial blood and tissue samples were collected periodically throughout the length of the study. Blood samples were drawn into EDTA-treated tubes. Lymph node biopsies were collected according to sterile surgical procedure and immediately transferred into tubes containing complete RPMI-10 on ice for transport. Blood and tissues were processed immediately. Complete blood counts were performed on EDTA-anticoagulated blood. Samples were analyzed with ProCyte Dx automated hematology instrument (IDEXX Laboratories, Westbrook, ME, USA), utilizing the parameters defined for baboons by the SNPRC. e-CHECK (XS) quality control (Sysmex) was analyzed each day at both normal and low levels. At D0, animals were challenged intravenously with 10^4^ tissue culture-infective dose (TCID_50_) of a cell-free inoculum stock SIVmac251 (*n*=3) or SIVbn-PBMC P4 s1 (*n*=4) diluted in sterile phosphate buffered saline (PBS, ThermoFisher).

### Viral nucleic acid isolation and quantification

Plasma viral RNA was isolated via the QIAamp Viral RNA Mini Kit (QIAGEN). Prior to isolation, plasma samples were spiked with a known quantity of Qβ bacteriophage (Attostar) to control for efficacy of extraction and amplification of recovered RNA. Cellular DNA was isolated via the Quick-DNA Miniprep Plus Kit (Zymo) from 1.0x10^6^ cells stored as a dry cell pellet at −80°C. Cell-associated RNA was extracted with the Direct-zol RNA Miniprep Plus kit (Zymo) from 1.0x10^6^ cells lysed in TRIzol reagent (ThermoFisher, Cat No: 15596018) and stored at −80°C. All isolated nucleic acids were stored at −80°C until the time of assay. Quantitative PCR (qPCR) for the detection of SIV DNA were prepared using the Platinum Quantitative PCR SuperMix-UDG (ThermoFisher) according to manufacturer’s recommendations. The levels of SIV gag DNA were represented as gag copies/10e6 cells, normalized against host cells Oncostatin M (OSM). Quantitative reverse transcription PCR (RT−qPCR) reactions were prepared using the RNA Ultra Sense One-Step Quantitative RT-PCR system (ThermoFisher) according to manufacturer’s recommendations. Plasma viral loads were determined by quantification of SIV gag. Qβ bacteriophage (internal control) was detected using primers and probe purchased from the manufacturer (Attostar LLC, St. Louis Park, MN). The concentration of SIVmac was determined using a standard curve generated from SIVmac genomic RNA. The sequences for all primers and probes are listed elsewhere (Obregon-Perko et al., 2018). All qPCR and RT−qPCR assays were run on an ABI 7500 system (ThermoFisher). Data was collected and analyzed using the accompanying SDS System Software.

### Statistics

Prism (GraphPad Software) was used to create figures and perform statistical analysis. The statistical test used for each experiment is noted in the figure legends. Significance was defined as a P value of <0.05.

## Results

### Generation and growth kinetics of baboon-adapted SIV

To generate a baboon-adapted SIV (SIVbn), we serially passaged SIVmac in baboon cells from STLV and SIV seronegative donors *in vitro*. In our first series, serial passage was done in PBMC from different donors at each passage (**Figures 1A, D**) with the goal of exposing the virus to selective pressure exerted by different host genetic backgrounds. The first passage was done in PBMC isolated from weanling baboons (about 8 m/o). These cells were susceptible to infection but grew the virus to a very low titer (**Figure 1A**). We observed that the age of the donor had a significant impact on the capacity of PBMC to support SIVmac replication (**Supplementary Figure 1**); that is, older baboons replicated SIVmac at higher levels than PBMCs from juveniles younger than 4 years of age in the absence of any stimulation (**Supplementary Figure 1A**). However, that difference between adults and juveniles disappeared when cells were stimulated with PHA (**Supplementary Figure 1B**), or when isolated CD4 cells were used for infection (**Supplementary Figure 1C**). To eliminate this age-dependent variability, baboon PBMC cultures were consistently stimulated with PHA. PBMC from juvenile baboons supported infection but viral replication kinetics during early PBMC passages were slow; remarkably, PBMC passages 4 and 5 showed a dramatic increase in replication kinetics, with viral loads peaking in these later passages about 7 d earlier than what was seen for passages 2 and 3 (**Figure 1A**).

In a new series, we repeated our PBMC passage to determine the changes resulting from repeatedly exposing the virus to selective pressures from the same host genetic background. Thus, we used cells from the same two baboon donors for each passage (**Figures 1B, D**). These animals were selected based on historical data showing their PBMC were more permissive for SIVmac growth relative to other donors we tested in our laboratory. SIVmac was also passaged in isolated CD4 cells from these same two donors to observe if the absence of other lymphocyte types present in PBMC populations would impact viral growth and adaptation. As expected, baboon CD4 cells were more permissive to infection, even at early passages (**Figure 1C**). Even so, there was still some evidence of adaptation as replication kinetics increased by passage 4. Surprisingly, we did not observe any evidence of viral adaptation during series 2 of PBMC serial passage, as earlier passages showed replication kinetics similar to the final passage.

We focused our attention on SIVbn-PBMC P4s1 along with SIVbn-CD4 P4 and designed an experiment that would address two questions about these viruses: (1) Will the observed adaptation of SIVbn-PBMC P4s1 and SIVbn-CD4 P4 be evident across different baboon donors? (2) Is serial passage in baboon CD4 cells, in the absence of other lymphocyte types, sufficient to confer enhanced replication kinetics in the mixed-lymphocyte environment of baboon PBMC? PBMC and CD4 cells were isolated from the same baboon donor, different from those used for serial passages, and infected contemporaneously with SIVmac, SIVbn-PBMC P4s1, or SIVbn-CD4 P4. SIVbn-PBMC P4s1 showed increased replication kinetics in baboon PBMC compared to the parental virus SIVmac (**Supplementary Figure 2A**). In contrast, SIVbn-CD4 grew relatively poorly in baboon PBMC. All viruses grew at similar rates and to similar titers in donor-matched CD4 cells, indicating differences in replication kinetics were not attributed to defects in viral replication (**Supplementary Figure 2B**). Instead, increased replication efficiency of SIVbn-PBMC s1 in baboon PBMC may be due to viral adaptation to mechanisms of restriction present in PBMC but absent in the permissive environment of isolated CD4 cells, whereas exposure and adaptation to only CD4-intrinsic mechanisms of viral restriction during serial passage in CD4 cells is insufficient to overcome restriction in PBMC, highlighting strong contributions from other cell types in exerting this immune pressure.

### Identification of changes to SIVbn genome

**Figure 2:**
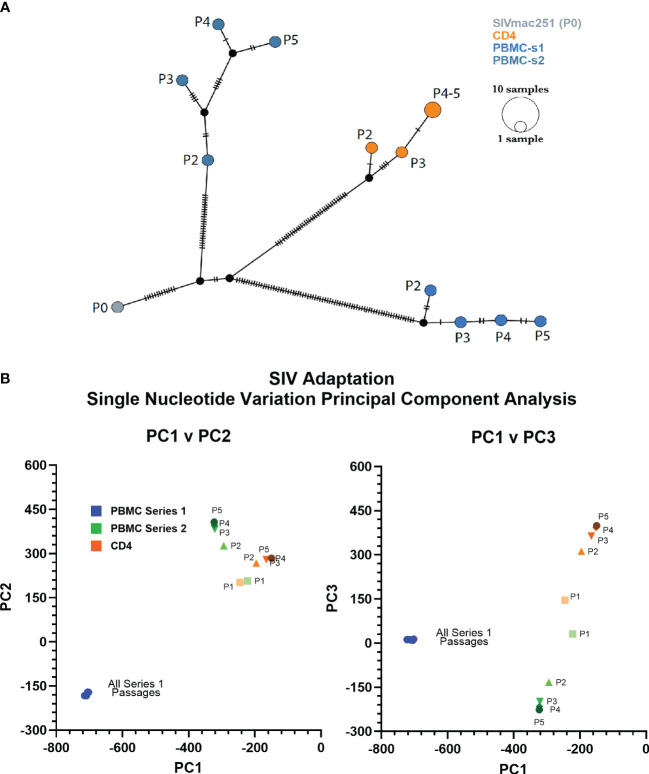
PBMC series 1 passage viruses are more divergent from SIVmac. **(A)** Haplotype network showing distance relationships between parental SIVmac251 (P0), PBMC-passaged viruses, and CD4-passaged viruses. Each tick represents a nucleotide variation that reached ≥50% in the viral population. Px indicates passage number. **(B)** Principal component analyses of the mutations accumulated by different viruses after each passage.

We used deep-sequencing to analyze the impact of serial passage on the SIVmac genome to understand how only SIVbn-PBMC from series 1 could demonstrate adaptation in PBMC. Data could not be obtained from passage 1 of PBMC series 1 and CD4 cells due to poor viral RNA recovery. However, sequencing data from the second passages indicated the viruses passaged in both PBMC series 1 were more divergent from SIVmac (P0) (**Figure 2**); overall, viruses from PBMC series 2 and CD4 cell passages, which both shared the same donors, were more closely related to each other and to SIVmac (**Figure 2B**). For all lineages, the largest change to the population occurred before passage 2 (P2), with little change to the viral population in subsequent passages; however, this early selection seemed stronger in the PBMC series 1 passages, as the second passage virus had the highest number of nucleotide variations that increased in frequency from SIVmac. This dramatic change to the viral population in PBMC P2 in series 1 suggests there was a strong unique positive selection event that occurred early during passage in this series and may have contributed to adaptation observed in later passages.

**Figure 3:**
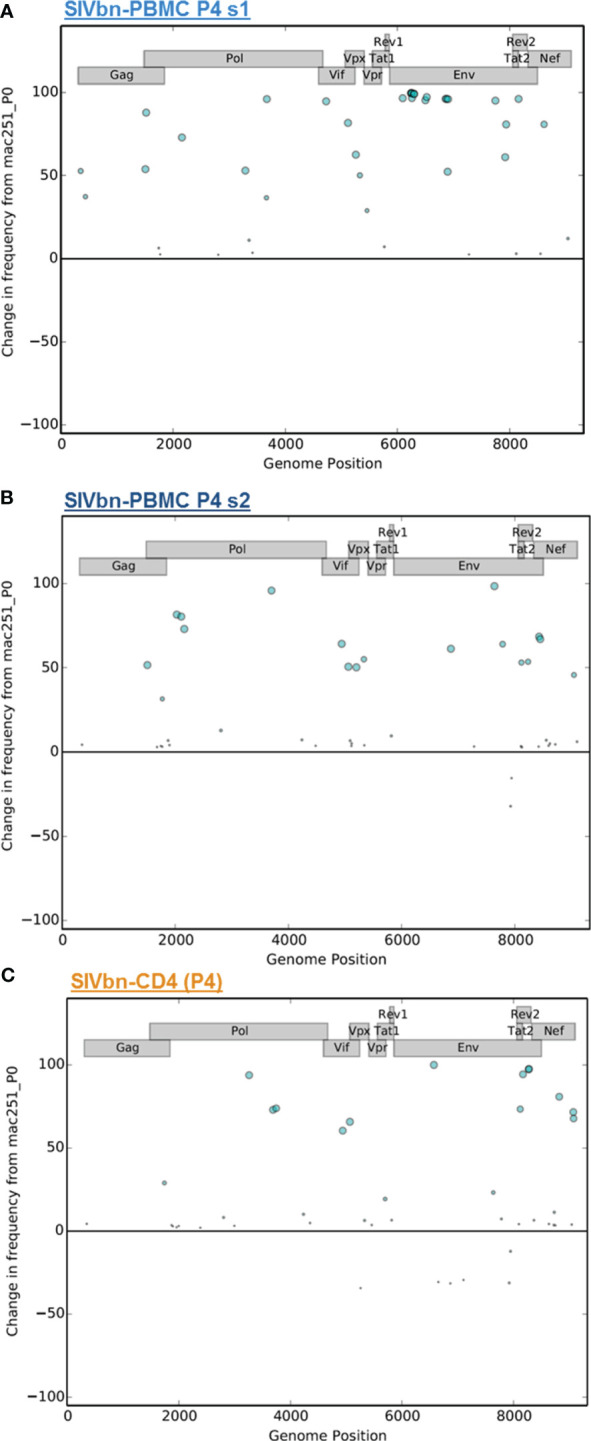
Distribution and change in frequency of non-synonymous changes across the SIVbn genomes. Vertical axis plots the percent change in frequency of a non-synonymous change in passage 4 viruses obtained from **(A)** SIVbn-PBMC s1, **(B)** SIVbn-PBMC s2, and **(C)** SIVbn-CD4 relative to SIVmac251 (P0). Circle placement on horizontal axis corresponds to location of change in genome; size is proportional to percent change in frequency.

We compared the abundance and distribution of non-synonymous changes across the viral genomes of SIVbn-PBMC s1, SIVbn-PBMC s2, and SIVbn-CD4 variants (**Supplementary Table 1**). Non-synonymous changes were seen across the genome for all viruses (**Figures 3A–C**). SIVbn-CD4 viruses accumulated a lower amount of non-synonymous changes (14 fixed mutations) than either SIVbn-PBMC s1 (29 fixed mutations) or SIVbn-PBMC s2 (17 fixed mutation) viruses. Unique to SIVbn-PBMC s1 viruses, many non-synonymous changes showed a nearly 100% change in frequency relative to SIVmac, indicating these variants were absent or present at very low levels in the parental virus but became nearly fixed in the SIVbn-PBMC s1 populations (**Figure 3A**). SIVbn-PBMC s2 and SIVbn-CD4 viruses, on the other hand, had fewer non-synonymous changes that showed over 90% change in frequency over SIVmac (**Figures 3B, C**). These findings were consistent with our hypothesis of a stronger selective pressure in the heterogeneous PBMC environment of series 1 passage, which may have selected for dominant variants capable of circumventing mechanisms of viral suppression in baboon PBMC.

Thus, serial passage in heterogeneous baboon PBMC, relative to isolated CD4 cells, exerted a strong selective pressure from mechanisms that require the action of other lymphocyte types, contributing to a higher number of fixed non-synonymous mutations in SIVbn-PBMC P4 s1. Also, within a homogenous genetic background, selective pressure by these other-than-CD4 lymphocytes leads to a different set of fixed mutations.

### SIV mutation by APOBEC3 enzymes

**Figure 4:**
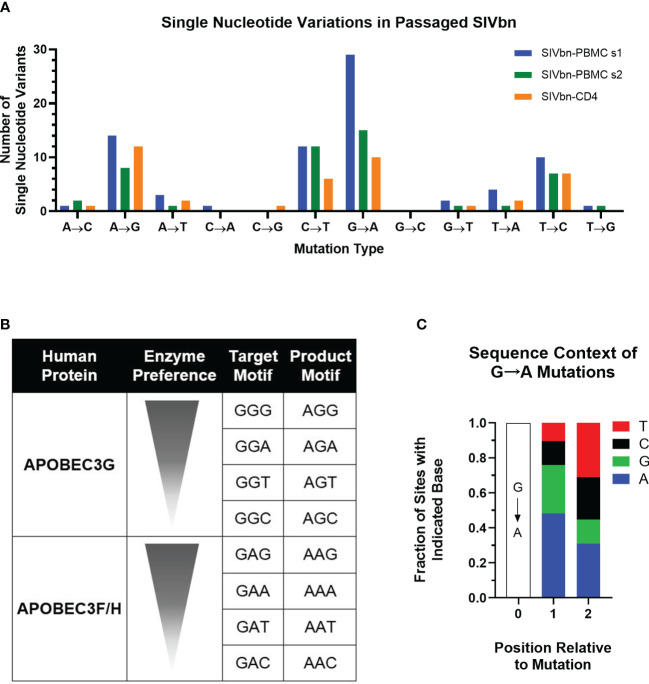
Analysis of single nucleotide variation (SNV) across the SIVbn genomes after 5 passages. **(A)** Vertical axis plots the number of SNV for SIVbn-PBMC series 1, SIVbn-PBMC series 2, and SIVbn-CD4 relative to SIVmac251. **(B)** Visual representation of human APOBEC preference for certain 3 nucleotides motifs, sorted within their groups, from most (top) to least (bottom) preferred. Adapted from Ebrahimi et al. **(C)** Global fraction of nucleotides for the G=>A variation of all 3 series.

When focusing on single nucleotide variants (SNV) that were present at frequencies below 25% in SIVmac251 and were found to be higher than 75% in the passaged viruses, we noted an overrepresentation of G>A mutations that suggests a possible role for APOBEC3 proteins in SIV adaptation (**Figure 4A**). APOBEC3 causes cytosine deamination in the negative strand of the HIV DNA during reverse transcription, which can be interpreted as a G>A mutation in the positive strand. There are two distinct footprints caused by different members of the APOBEC3 family (**Figure 4B**). We analyzed the trinucleotide context of the G>A mutations present in the passaged viral RNA and discovered that, as found for rhesus macaques, the APOBEC3D/F/H GA>AA footprint is dominant in baboons (**Figure 4C**). It is worth noting that our analysis was done on viral RNA sequences, not the integrated proviral DNA. APOBEC3 proteins can cause integrated sequences to become completely defective, and therefore, incapable of producing viable viral particles. Thus, our findings suggest that baboon APOBEC3 enzymes have primarily introduced sublethal levels of mutations impacting *in vivo* replication by facilitating SIV evolution.

### SIVbn co-receptor usage

**Figure 5:**
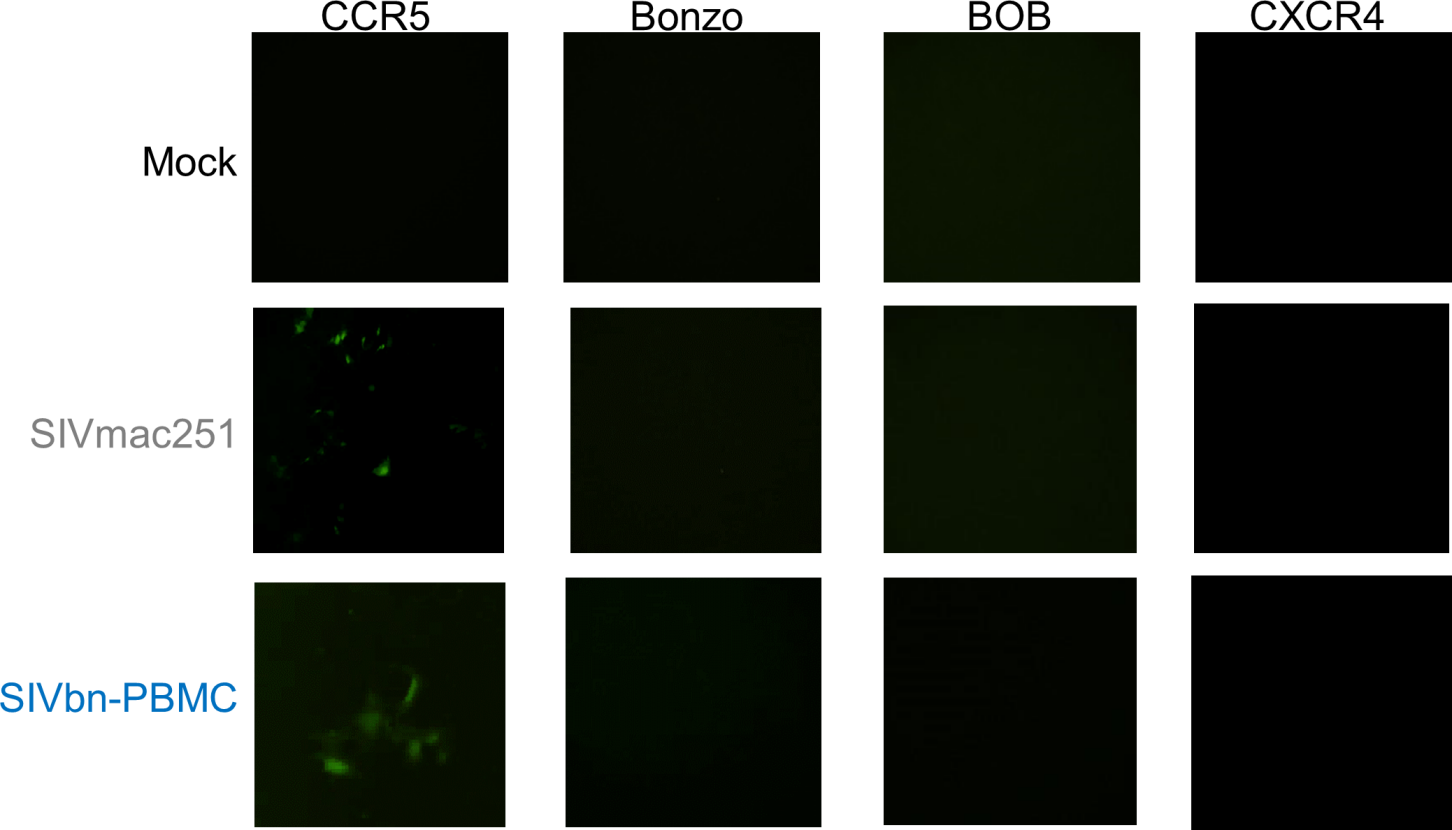
SIVbn-PBMC Is CCR5-Tropic. GHOST indicator cell lines expressing human CD4, one of indicated co-receptors (CCR5, Bonzo, BOB, or CXCR4), and a green fluorescent protein (GFP) construct under control of Tat were used to assess co-receptor tropism. Cells were mock-treated (top), infected with SIVmac251 (middle), or infected with SIVbn-PBMC series 1 (bottom) at a M.O.I. of 10^4^ g.e./cell for 2 hr. Images taken 48 hr post-infection.

Considering our previous data demonstrating chemokine-mediated inhibition of SIVmac entry in baboon PBMC (Obregon-Perko et al., 2018), we hypothesized variations in *Env* may have been positively selected for during PBMC series 1 passage to overcome CCR5 blockade. The most obvious way to circumvent reduced availability of CCR5 is to enter target cells through another co-receptor. Thus, we asked if SIVbn-PBMC s1 had undergone a co-receptor switch or gained the ability to use additional co-receptors. Toward this end, we performed infections in indicator cell lines that express CD4 and one of various co-receptors. These cells also stably express a green fluorescent protein (GFP) construct under the control of the viral protein Tat, so fluorescence can be used as readout for infection. As expected, SIVmac only generated GFP signal in CCR5^+^ cells and was unable to infect cells through Bonzo, BOB, or CXCR4 (**Figure 5**). Similarly, GFP signal was only obtained from CCR5^+^ cells exposed to SIVbn-PBMC s1, indicating that this virus remained CCR5-tropic even after serial passage.

To confirm CCR5 as the principal co-receptor used by SIVbn-PBMC s1, we treated baboon CD4 cells with the CCR5 antagonist maraviroc before and during infection. Treatment with maraviroc reduced SIVmac and SIVbn-PBMC s1 viral loads to nearly undetectable levels, indicating both viruses are highly dependent on CCR5 for viral entry (**Supplementary Figure 3A**). Since a high concentration of maraviroc (1 µm) was used to maximally inhibit infection, we wondered if resistance to CCR5 blockade in SIVbn-PBMC P4 s1 might only be revealed at lower concentrations of the drug. Thus, we exposed baboon CD4 cells to 10-fold serial dilutions of maraviroc during SIVmac and SIVbn-PBMC P4 s1 infection. SIVmac viral loads could be reduced in baboon CD4 cells by nearly 50% at 1 nM maraviroc (**Supplementary Figure 3B**). SIVbn-PBMC P4 s1 was less sensitive to lower doses of maraviroc, requiring about 10 nM to reduce viral loads by 50%; however, higher concentrations could reduce viral loads of both viruses to similar levels (**Supplementary Figures 3A, B**), suggesting SIVmac and SIVbn-PBMC P4 s1 have a weak ability to use drug-bound receptors for entry. Taken together, these studies support a model whereby serial passage of SIVmac in baboon PBMC selected for viral variants that may have adapted to chemokine-mediated CCR5 blockade by more efficiently using unoccupied CCR5.

### Generation of single mutant SIV genomes

**Figure 6:**
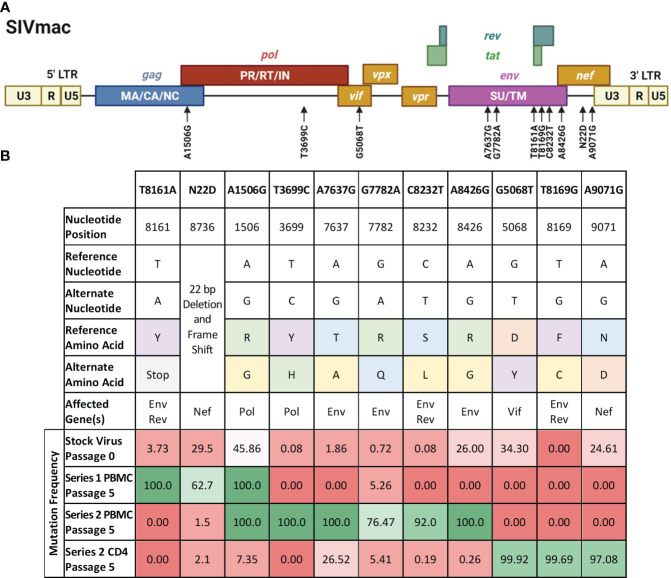
Mutations of interest fixed in the SIVbn genomes. **(A)** Schematic of the mutations of interest (black arrows) relative to the SIVmac genime. Polyproteins are indicated within their respective boxes. LTR, long terminal repeat; MA, matrix; CA, capsid; NC, nucleocapsid; PR, protease; RT, reverse transcriptase; IN, integrase; SU, surface protein; TM, transmembrane protein. Figure adapted from the HIV Sequence Compendium 2018 (Foley et al). **(B)** List of single mutant viruses. Header row represents designation of each individual mutations of interest identified by filtering scheme. Amino acid classification is color coded as follows, purple: aromatics; green: positively charged; orange: negatively charged; blue: polar; yellow: nonpolar; grey: premature stop codon. Mutation frequency is percent prevalence of alternate allele. Red shading represents mutation frequency <50%, while green shading represents >50% prevalence.

We sought to study the contribution of some of the passage-acquired SIV mutations to the overall fitness of the baboon-adapted viruses. RNA deep-sequencing of all three lineages of baboon-adapted SIVmac revealed over 400 mutations present in the various passages. These included synonymous and non-synonymous mutations, as well as changes to non-coding regions. We created a filtering scheme to identify the mutations that were most likely to have had a significant impact on SIV replication kinetics. First, we opted to only consider non-synonymous mutations (247 in total, **Supplementary Table 1**), as they changed the viral protein sequences. Next, we focused on mutations with a divergent bias, that is mutations present in only one passage series (61 in total). Finally, to have the best chance of identifying a single mutation that can substantially impact viral replication, we opted for mutations that cause a significant amino acid change (9 in total), for example, a change from arginine, a basic hydrophilic amino acid, to glycine, a hydrophobic alkyl amino acid. We also included two additional mutations that resulted in significant truncations in their respective viral proteins. The first was a mutation resulting in a premature stop codon in the region of Env that overlaps with Rev. The second is a 22 base pair deletion and resulting frameshift in Nef. All mutations of interest (11 in total) are summarized in **Figure 6**.

**Figure 7:**
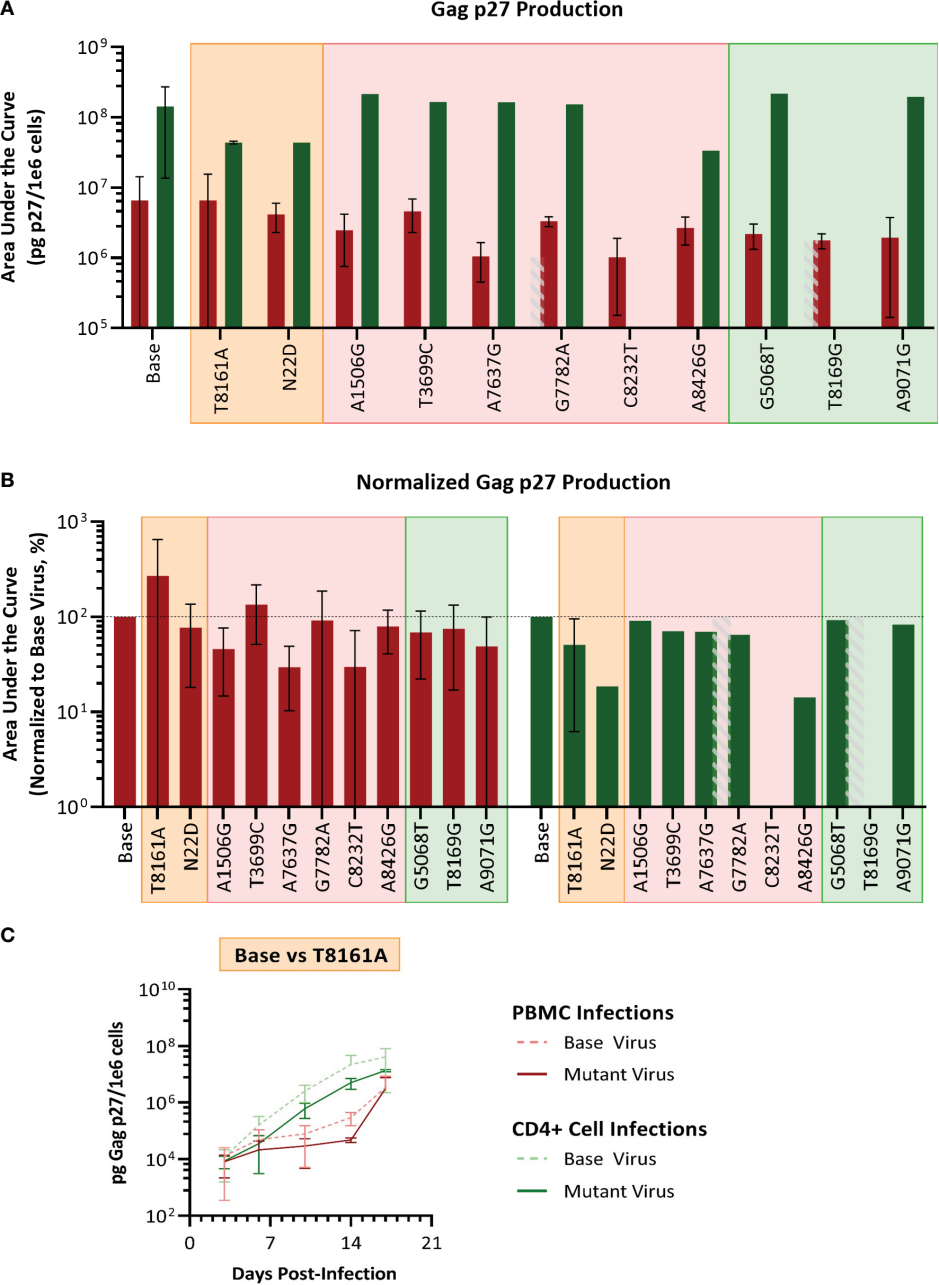
Analysis of single mutant virus replication kinetics. PHA-stimulated baboon PBMC (red) or isolated CD4+ cells (green) were infected with SIVmac239 (base virus) or mutant SIV at an M.O.I. of 100 vp/cell. **(A)** Replication efficiency analysis; bars represent the production of Gag p27 as area under the curve (AUC) from D3–D17 post-infection. **(B)** Normalized AUC analysis as a percent of their base virus infections in their respective cell type. Background colors represent mutation series 1 PBMC (orange), series 2 PBMC (pink), or Series 2 CD4 (green); the grey striped bar is a placeholder for a discontinued infection, where AUC could not be calculated. **(C)** Example of replication curves for the T8161A mutant virus.

We selected SIVmac239, a pathogenic molecular clone derived from the biological isolate SIVmac251 (Kestler et al., 1990) as the base virus. Recombinant SIVmac239 viruses were generated in HEK cells that included one each of the 11 mutations selected. Each mutant virus was used to infect PBMC and isolated CD4 T cells, which were obtained from the same baboon donors used for PBMC series 2 and CD4. The replication kinetics of each mutant virus were compared to the ones generated by the parental virus SIVmac239 by plotting the concentration of SIV p27 versus days post-infection graphs and calculating the area under the curve (AUC) (**Figure 7**). We found that the mean AUC for all the infections performed with the mutant viruses in CD4+ cells (1.37x10^8^ pg SIV p27/10^6^ cells) was significantly higher (p=0.0008, paired t-test) than infections in PBMC with the same mutant viruses (3.22x10^6^ pg SIV p27/10^6^ cells) (**Figure 7A**). When the AUC was normalized to the base virus SIVmac239, a majority of both the PBMC and CD4+ cell infections performed below baseline (**Figure 7B**). For the PBMC infections, mutation T8161A exhibited the greatest increase in replication efficiency over base (270.2%), though there was a high degree of variability amongst the replicates, and therefore the change was not statistically significant. Mutations A7637G and C8232T infections exhibited the lowest normalized replication efficiency in PBMC and were both well below baseline (29.6% and 29.7%, respectively). All CD4+ cell infections with single-mutated viruses all performed below baseline (range: 14.2–91.1%).

Thus, single mutant viruses did not reproduce the increased fitness observed for the complex swarm viruses generated in each series.

### *In vivo* challenge with SIVbn

We finally tested the hypothesis that SIVbn, an SIVmac adapted to grow in baboon primary cells, would overcome the viral growth restrictions presented by baboons in nature. Previous studies have demonstrated that the ability of an animal’s PBMC to support SIVmac replication *in vitro* is generally a reflection of what happens in the same animal *in vivo* (Lifson et al., 1997; Goldstein et al., 2000). Thus, to determine whether SIVbn-PBMC series 1 or series 2 was best suited for *in vivo* experimentation, we performed *ex vivo* baboon PBMC infections (*n*=7) with passage 4 of both series strains, as well as the parental virus SIVmac251, using cells from the same baboons that would be used for *in vivo* infections. After 10 days in culture, baboon PBMC infected with SIVbn-PBMC s1 virus generally had higher viral loads compared to the other viruses (**Supplementary Figure 4**). As such, SIVbn PBMC P4 s1 was selected for *in vivo* challenge.

**Figure 8:**
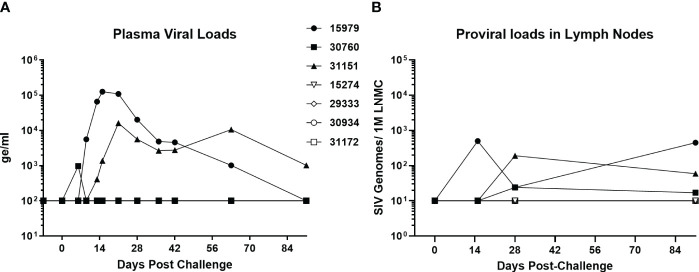
SIV-bn PBMC P4 s1 does not reach detectable viral loads in baboons *in vivo*. Baboons were challenged with 10,000 TCID_50_ of SIVmac251 (n=3, solid markers) or SIVbn-PBMC s1 (n=4, open markers), delivered by the intravenous route in a 1 ml volume. Blood samples **(A)** and lymph node biopsies **(B)** were collected at regular intervals and SIV RNA and integrated DNA were quantified by real-time RT-PCR and real time PCR, respectively. Samples from baboons infected with SIVbn-PBMC s1 were below the limit of detection of the assays.

A group of four baboons was infected with SIVbn-PBMC s1, while a second group of three animals received SIVmac251. The viral dose was the same 10^4^ TCID_50_ infectious dose, delivered in the same 1ml volume and by IV injection. Two of the three SIVmac251 challenged baboons had detectable viral loads by day 13, while the third baboon in the group exhibited an early peak viral load at day 6 that declined to undetectable levels (<100 genome equivalent/ml) by day 10 (**Figure 8A**). This pattern was also evident for proviral loads in lymph node mononuclear cells (**Figure 8B**). Interestingly, though SIVbn-PBMC P4 s1 was able to replicate more efficiently in baboon PBMC *ex vivo*, none of the corresponding *in vivo* inoculations resulted in detectable viral loads in peripheral blood or lymph node biopsies throughout the course of the study (90 days). Thus, *in vitro* adaptation of SIVbn-PBMC P4 s1 did not confer adaptation *in vivo* but, instead, likely introduced mutations deleterious for *in vivo* replication.

## Discussion

The AIDS pandemic is the consequence of zoonotic transmissions of SIVs infecting chimpanzees and gorillas in sub-​Saharan Africa. Adaptation of an SIV to a NHP species has been the outcome of viral protein and host factor interactions that resulted in nonpathogenic chronic SIV infections of more than 40 NHP species (Sauter and Kirchhoff, 2019). However, baboons have remained SIV-free for millennia. Understanding the basis for this natural baboon immunity to SIV infection may help to develop new preventive and therapeutic approaches. Previous work from our group has shown the CD4 cell environment of baboons is highly permissive to SIVmac infection *ex vivo*, suggesting intrinsic antiviral factors that typically impose barriers to cross-species transmission (e.g. TRIM-5α), likely do not play a strong role in the observed SIV immunity in baboons. By contrast, viral replication is significantly dampened by other lymphocyte types in SIV-naïve baboon PBMC relative to rhesus macaques (Obregon-Perko et al., 2018). In this work, we sought to confirm if mechanisms of SIVmac restriction observed in baboon PBMC were unique to the mixed-cell environment and to uncover the viral gene products targeted by these mechanisms by serially passaging SIVmac in the highly permissive environment of CD4 cells and in the less-permissive environment of PBMC, and then analyzing changes to the viral genome. We designed three different passage scenarios: PBMC series 1, using different donors for each passage to maximize host genetic pressure on the virus; PBMC series 2, using the same donors for each passage, to maintain a constant and invariable host genetic pressure on the virus; and CD4 series, using isolated CD4 cells derived from the same donors as series 2, to identify the type of pressure produced by non-CD4 cells in series 2.

Growth in each of these cell systems generated viral stocks with different genotypes relative to the original viral population (**Supplementary Table 1**; **Figures 2**, **3**). SIVbn-PBMC P4 s1 acquired more nucleotide variations by the second passage than what was seen for SIVbn-PBMC P4 s2 or SIVbn-CD4 P4. In addition, many non-synonymous changes became fixed in the PBMC series 1 passage populations by the final passage; fixed changes were less frequent in PBMC series 2 and CD4 passage populations, but these changes were also different from each other, even when the virus replicated in cells from the same donors. All of which is suggestive of a stronger immune pressure in PBMC series 1 passages, derived from fourteen donors across five passages, compared with series 2, which used the same two donors for each of the five passages. Thus, differences in genetic background of the baboon PBMC donors may have contributed to the distinct outcomes of the PBMC serial passages. PBMC from baboon weanlings were largely refractory to SIVmac infection, and so their use in the first passage of series 1 may have also contributed to a strong positive selection event that did not occur in series 2 because older juvenile baboons were used to initiate the passages. Thus, we hypothesized conditions in the PBMC series 1 passages favored early selection for pre-adapted genotypes that acquired further changes in subsequent passages that together conferred a growth advantage in baboon PBMC. Indeed, SIVbn-PBMC P4 s1, but not SIVbn-CD4, demonstrated increased replication kinetics in baboon PBMC. On the other hand, SIVbn-CD4 and SIVbn-PBMC P4 s1 had similar infectivity in isolated CD4 cells. Thus, the increased replication kinetics observed for SIVbn-PBMC P4 s1 in baboon PBMC is likely not due to adaptation to CD4-intrinsic mechanisms, such as intracellular restriction factors.

Through deep-sequencing, we identified genetic determinants that could be responsible for the adaptation of SIVbn-PBMC to baboon PBMC. Eleven candidate non-synonymous nucleotide variations that increased in frequency during PBMC passage mapped to *Env*; three of these fell within or very close to the V3 loop. Changes in co-receptor tropism or affinity are typically attributed to sequence variations within this region (Hwang et al., 1991; Ivanoff et al., 1992; Trujillo et al., 1996). We previously identified one mechanism of SIVmac restriction in baboon PBMC that involves increased production of CCR5 ligands (CCL3, CCL4, and CCL5) that interfere with viral entry (Obregon-Perko et al., 2018). Therefore, we speculated that variations to the V3 loop of *Env* may have arisen in SIVbn-PBMC s1 to circumvent co-receptor blockade by CCR5-binding chemokines. In support of this notion, resistance to small molecule CCR5 inhibitors has been reported for HIV infection *in vitro* (Moore and Kuritzkes, 2009). The principal pathways of viral resistance typically involve use of alternate co-receptors, continued use of inhibitor-bound CCR5, or more efficient use of unoccupied CCR5 (Pugach et al., 2007; Westby, 2007). Infections in co-receptor indicator cell lines confirmed there was no generation or expansion of variants during PBMC series 1 passages capable of using other co-receptors (**Figure 5**). We used maraviroc to uncover changes in sensitivity to CCR5 blockade in SIVbn-PBMC P4 s1. Here, we saw neither SIVmac nor SIVbn-PBMC P4 s1 had the ability to use drug-bound CCR5 to infect target cells. However, a dose-response analysis revealed SIVbn-PBMC P4 s1 was more resistant than SIVmac to lower doses of maraviroc; to achieve 50% reduction in viral loads, SIVbn-PBMC P4 s1 required as much as 10 times more drug than SIVmac (**Supplementary Figure 3**). Thus, as one mechanism of adaptation, we propose SIVbn-PBMC P4 s1 more efficiently uses unoccupied CCR5 compared to SIVmac, which explains why resistance to maraviroc is only seen at doses that do not completely occupy all co-receptors. Because CCR5-binding chemokines are present at lower levels in the absence of CD8 T and NK cells (Obregon-Perko et al., 2018), these variations in receptor engagement may not confer enhanced replication efficiency in isolated CD4 cells.

Resistance to CCR5 blockade can also be conferred by changes outside the V3 loop (Ogert et al., 2008; Espy et al., 2017). Interestingly, the SIVbn-PBMC s1 populations had non-synonymous changes of high frequency in gp41, the portion of the *Env* that regulates membrane fusion (Wilen et al., 2012). One change resulted in a premature stop codon in the cytoplasmic domain of *Env*. SIV variants with a truncated cytoplasmic tail incorporate higher amounts of Env into nascent virions and have been shown to have a higher infectivity (Zingler and Littman, 1993). This may be due to increased affinity for entry receptors or facilitated viral spread through syncytium formation (Zingler and Littman, 1993). These proposed mechanisms may be acting alone or in concert to more efficiently use available co-receptors when faced with chemokine-mediated CCR5 blockade.

Although increased production of CCR5 ligands plays an important role in the suppression of SIVmac in baboon PBMC, restriction is likely mediated by multiple mechanisms. Nucleotide variations were also seen outside of *Env* in SIVbn-PBMC and cannot be immediately dismissed as contributors to the observed adaptation. Changes to the viral accessory proteins Vif, Vpx, and Nef were also seen during serial passage in baboon PBMC, which could be at least partially responsible for the observed adaptation of SIVbn-PBMC s1. Vif and Vpx can target various intracellular host restriction factors, such as APOBEC3G and SAMHD1, for proteasomal degradation (Marin et al., 2003; Sheehy et al., 2003; Yu et al., 2003; Ahn et al., 2012; Hofmann et al., 2012). Nef is known to downregulate surface proteins, such as tetherin and MHC-I (Greenberg et al., 1998; Le Gall et al., 2000; Zhang et al., 2011), the latter being a sophisticated process to balance evasion from CD8 T cells with detection by NK cells. Curiously, the change to Nef was one of the nucleotide variations that showed a more gradual increase in frequency; its sudden increase in frequency by passage 4 was mirrored by a jump in replication kinetics of this same passage during growth in PBMC series 1 (**Figure 1A**). While we have observed intracellular restriction factors and memory CTL responses do not play a significant role in the observed suppression of SIVmac in naïve baboon PBMC, changes to these viral proteins in SIVbn-PBMC s1 may have occurred to overcome yet unidentified or uncharacterized mechanisms of viral suppression in baboon PBMC.

Experiments performed with single mutant viruses suggested that the observed increases in fitness of SIVbn-PBMC s1 and SIVbn-CD4 were the result of contributions from several different variants, as the single mutant viruses that we generated had similar fitness as the parental SIVmac239 virus (**Figure 7**). This work highlights the importance of considering group effects and the potential impact of even low-frequency variants. In support of this model, we observed early selection of variants with changes to *Env* during PBMC series 1 passage. However, a significant increase in replication kinetics was not seen until later passages and mirrored nicely with a dramatic rise in the frequency of a *Nef* mutation, suggesting cooperation with late emerging variants may be required. Evaluating mutations that showed a more gradual increase in frequency should be further explored.

One critical question that needed to be addressed was whether this increase in baboon PBMC replication by SIVbn-PBMC s1 meant increased pathogenicity *in vivo*. Thus, we challenged baboons with SIVbn-PBMC P4 s1 or SIVmac 251. Remarkably, all animals challenged with SIVbn-PBMC P4 s1 had undetectable viral loads throughout the course of the study, even though cells from the same donors grew the virus *ex vivo* to levels that often exceeded those of SIVmac (**Supplementary Figure 4**). Thus, mutations that may have conferred adaptation *ex vivo* were detrimental to fitness *in vivo*. We hypothesize the identified deletions in Nef could be at least partially responsible for the altered viral dynamics *in vivo*, as the importance of Nef in reaching viremia and AIDS in rhesus macaques infected with SIVmac239 has previously been established (Kestler et al., 1991). As mentioned earlier, Nef mediates immune evasion in various ways, including MHC-I, CD3, CD4, and CD28 downregulation, suppression of Ig class-switching, and exclusion of the host restriction factors (tetherin, SERINC5) from nascent virions that impede viral entry. The flexible internal loop region of Nef (amino acids 182–199) serves as an adaptor for endocytosis of a number of transmembrane proteins by stabilizing interactions between the cytoplasmic domains of cellular cargo proteins and subunits of either the adaptor protein (AP)-1 or AP-2 complexes (Tavakoli-Tameh et al., 2020); AP complexes are heterotetrameric protein complexes that mediate intracellular membrane trafficking along endocytic and secretory transport pathways (Park and Guo, 2014). Mutation N22D is a 22 base pair deletion in Nef resulting in a frameshift and premature stop codon, shortening the protein from 264 amino acids in length to just 138 amino acids, and thus potentially eliminating the internal flexible loop and all its associated biological functions. In cell culture, the necessity of Nef is variable depending on the approach: in primary T cell or macrophage cultures infected without exogenous stimulation, Nef is required for productive viral replication (Munch et al., 2007), but in certain immortal cell lines and activated primary cells, functional Nef has little to no impact on replication (Miller et al., 1994). Since our *ex vivo* passages were performed in PHA-activated cells, the need for an active Nef protein may have been unnecessary; on the contrary, it led to the generation of a virus highly adapted to grow in cells from animals with different genetic backgrounds, but incapable to establish a productive infection *in vivo*.

Lentiviruses like HIV and SIV have notoriously plastic genomes and have co-evolved with their hosts for perhaps 5 to 10 million years (Compton et al., 2013). Understanding adaptation events to new host species may provide key insights into innate defense mechanisms and viral dependencies on cellular factors (Sauter and Kirchhoff, 2019). Baboons have been able to prevent SIVs from jumping into their genomes for thousands of years, even when they are evolutionary as closely related to sooty mangabeys, a natural non-pathogenic SIV host, and rhesus macaques, a non-SIV host that develops immunodeficiency when experimentally infected, as humans are to chimpanzees (Shao et al., 2023). Attempts to adapt HIV and SIV to baboons *in vivo* have been tried before, particularly with HIV-2, a close relative to SIVsmm from sooty mangabeys, by serial passages of strains of HIV-2, using blood and bone marrow samples obtained during the acute phase of infection; however, the virus used was either a dual tropic HIV-2 (Locher et al., 2003b), or the virulence of a virus was lost after further passaging in a new animal (Locher et al., 2001). Although it is questionable to extrapolate *ex vivo* SIV replication fitness to *in vivo* infectivity, some scientists have found correlations for SIVmac and rhesus macaques (Lifson et al., 1997; Goldstein et al., 2000), while others working with SIVsmm, the root source of SIVmac (Apetrei et al., 2005), and rhesus macaques have not (Gautam et al., 2007). This study extends this controversy as the clear outcome of *ex vivo* adaptation of SIVbn to grow in stimulated baboon PBMCs resulted in total lack of *in vivo* infectivity (**Figure 8**), whereas the capacity of SIVmac to replicate *ex vivo* in baboon PBMC (**Supplementary Figure 4**) was replicated in the *in vivo* replication patterns of SIVmac-infected baboons (**Figure 8**).

In conclusion, we have shown serial passage in baboon PBMC, but not in isolated CD4 cells, selects for viral variants adapted to overcome mechanisms of restriction unique to the mixed-cell environment of baboon PBMC and variable among host genetic backgrounds. These viral variants, however, are unable to establish a productive infection in live baboons, even when inoculated in the systemic circulation, suggesting that the mechanisms of baboon natural immunity to SIV infection are complex and need to be studied in the context of the whole organism. In this manuscript, we limited our studies mostly to the viral side of the virus-host interaction conflict. In this artificial cell culture environment, antiviral molecules that are produced at the tissue level or by fast-acting cellular components of the baboon innate immune system that are either in low numbers or absent in the PBMC environment, may not be present, therefore not exerting a selective pressure on the adapting virus that would improve *in vivo* adaptation. We are completing *in vivo* studies in which we are interrogating different cellular components of the baboon immune system to identify the mechanisms that have allowed this NHP species to evade or contain chronic SIV infections for thousands of years.

## Data availability statement

The raw data supporting the conclusions of this article will be made available by the authors, without undue reservation.

## Ethics statement

The animal study was approved by Texas Biomedical Research Institute Institutional Animal Care and Use Committee (IACUC). The study was conducted in accordance with the local legislation and institutional requirements.

## Author contributions

VO-P: Formal analysis, Investigation, Methodology, Writing – original draft, Writing – review & editing. AM: Formal analysis, Investigation, Methodology, Writing – original draft, Writing – review & editing. JL: Formal analysis, Investigation, Methodology, Writing – review & editing. VH: Formal analysis, Investigation, Methodology, Writing – review & editing. DE: Formal analysis, Investigation, Methodology, Writing – review & editing. LP: Formal analysis, Methodology, Writing – review & editing. JC: Methodology, Writing – review & editing. GP: Formal analysis, Investigation, Writing – review & editing. LG: Conceptualization, Data curation, Formal analysis, Funding acquisition, Investigation, Methodology, Project administration, Resources, Supervision, Validation, Writing – original draft, Writing – review & editing.

## Supplementary material

Supplementary Figure 1 Age impacts the outcome of SIVmac infection in baboon PBMC. PBMC were isolated from juvenile (light blue, n = 6) and adult (dark blue, n = 6) baboon donors. PBMC were cultured for 48 hr in **(A)** RPMI-10 or **(B)** RPMI-10 containing PHA before infection. **(C)** CD4 cells were sorted from PBMC then cultured for 48 hr in RPMI-10 before infection. All PBMC and CD4 cells were infected with SIVmac at a M.O.I. of 100 g.e./cell. Viral loads were quantified by measuring p27 in the supernatant by Luminex. In all panels, each curve represents one animal. Repeated measures two-way ANOVA was used for statistical analysis (****P ≤ 0.0001).

Supplementary Figure 2 SIVmac passaged in baboon PBMC, but not in CD4 cells, demonstrates adaptation for growth in Baboon PBMC. **(A)** PBMC and **(B)** CD4 cells were isolated from the same baboon donor and cultured for 48 hr in RPMI-10. Cells were infected with SIVmac251 (grey), SIVbn-PBMC P4S1 (blue), or SIVbn-CD4 (P4) (orange) at a M.O.I. of 100 g.e./cell. Viral concentrations were determined by measuring SIV p27 in the supernatant by Luminex. Data shown represents the median (n = 5). t-test was used for statistical analysis (*P ≤ 0.05).

Supplementary Figure 3 SIVbn-PBMC P4 s1 has reduced sensitivity to CCR5 blockade. CD4 cells were isolated from baboon PBMC and cultured for 48 hr in RPMI-10. **(A)** CD4 cells were incubated with PBS (Untreated) or 1 µM maraviroc then infected with SIVmac251 or SIVbn-PBMC at a M.O.I of 0.01. PBS or maraviroc was supplemented in media during culture. Bar graph displays p27 values normalized to the untreated condition at 14 d.p.i. (mean ± SEM) (n = 2). **(B)** CD4 cells were infected with SIVmac251 or SIVbn-PBMC at a M.O.I of 0.01. Media was supplemented with PBS or one of 10-fold serial dilutions of maraviroc (0.1 nm – 1 µM). Dose-response curve is shown as linear regression on log-transformed maraviroc concentrations against p27 values normalized to untreated condition at 10 d.p.i. Bars indicate SEM (n = 2). All viral loads were determined by measuring p27 in the supernatant by Luminex.

Supplementary Figure 4 SIVbn-PBMC P4 s1 has the highest replication efficiency in baboon PBMC. PBMC from seven naïve baboons were infected *ex vivo* with SIVmac251, or the baboon PBMC-adapted SIVbn-PBMC P4 s1 and PBMC P4 s2. SIVp27 concentration in the supernatant was quantified with a Luminex assay.
