# Supplementary figures and images for: Adaptation of SIVmac to baboon primary cells results in complete absence of in vivo baboon infectivity
Source: Front Cell Infect Microbiol. 2024 Jun 28;14:1408245. doi: 10.3389/fcimb.2024.1408245 (PMC11239360; doi:10.3389/fcimb.2024.1408245)

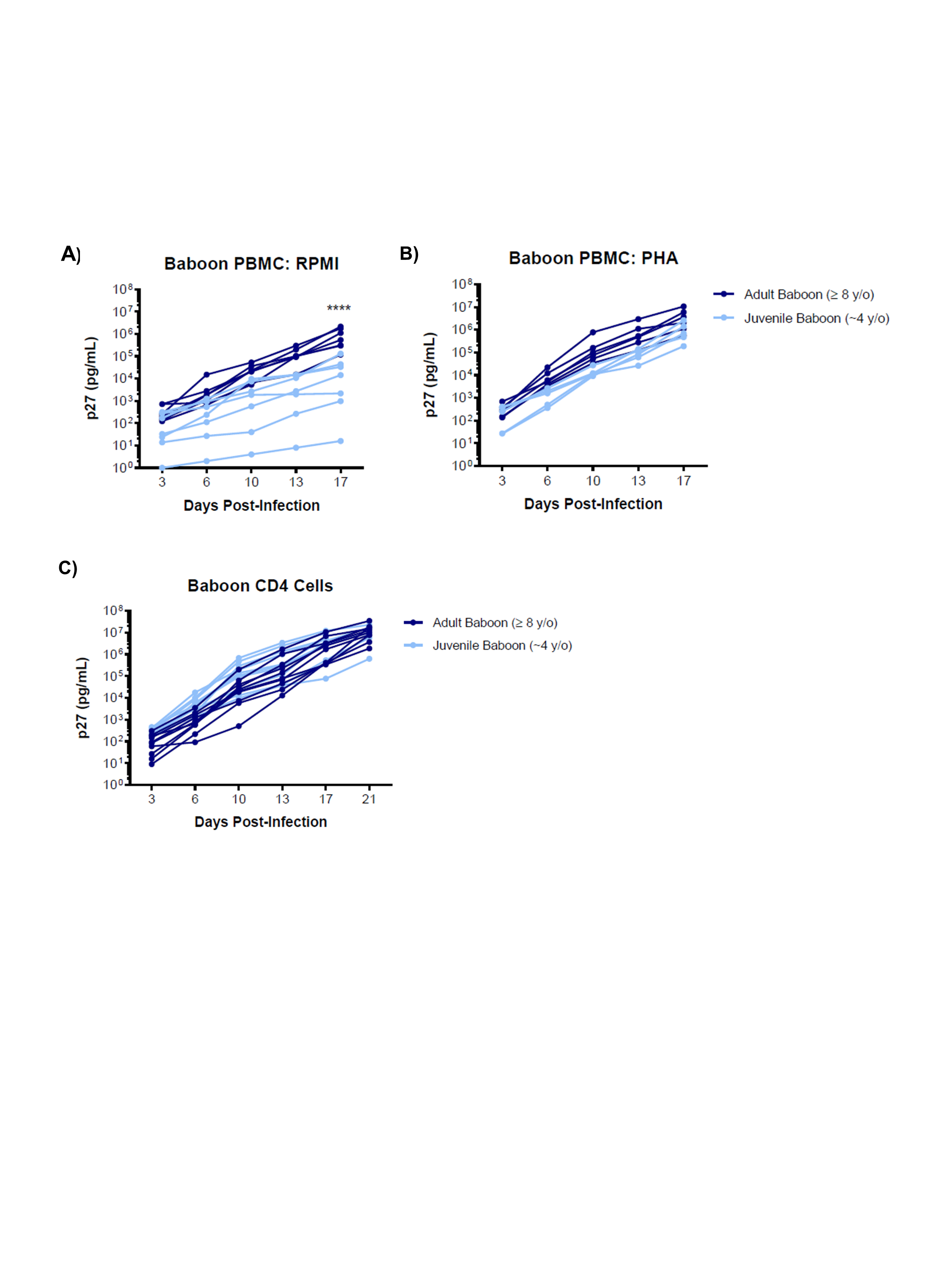

Supplement: Supplementary Figure 1 — Age impacts the outcome of SIVmac infection in baboon PBMC. PBMC were isolated from juvenile (light blue, n = 6) and adult (dark blue, n = 6) baboon donors. PBMC were cultured for 48 hr in (A) RPMI-10 or (B) RPMI-10 containing PHA before infection. (C) CD4 cells were sorted from PBMC then cultured for 48 hr in RPMI-10 before infection. All PBMC and CD4 cells were infected with SIVmac at a M.O.I. of 100 g.e./cell. Viral loads were quantified by measuring p27 in the supernatant by Luminex. In all panels, each curve represents one animal. Repeated measures two-way ANOVA was used for statistical analysis (****P ≤ 0.0001). [file Image_1.tif]

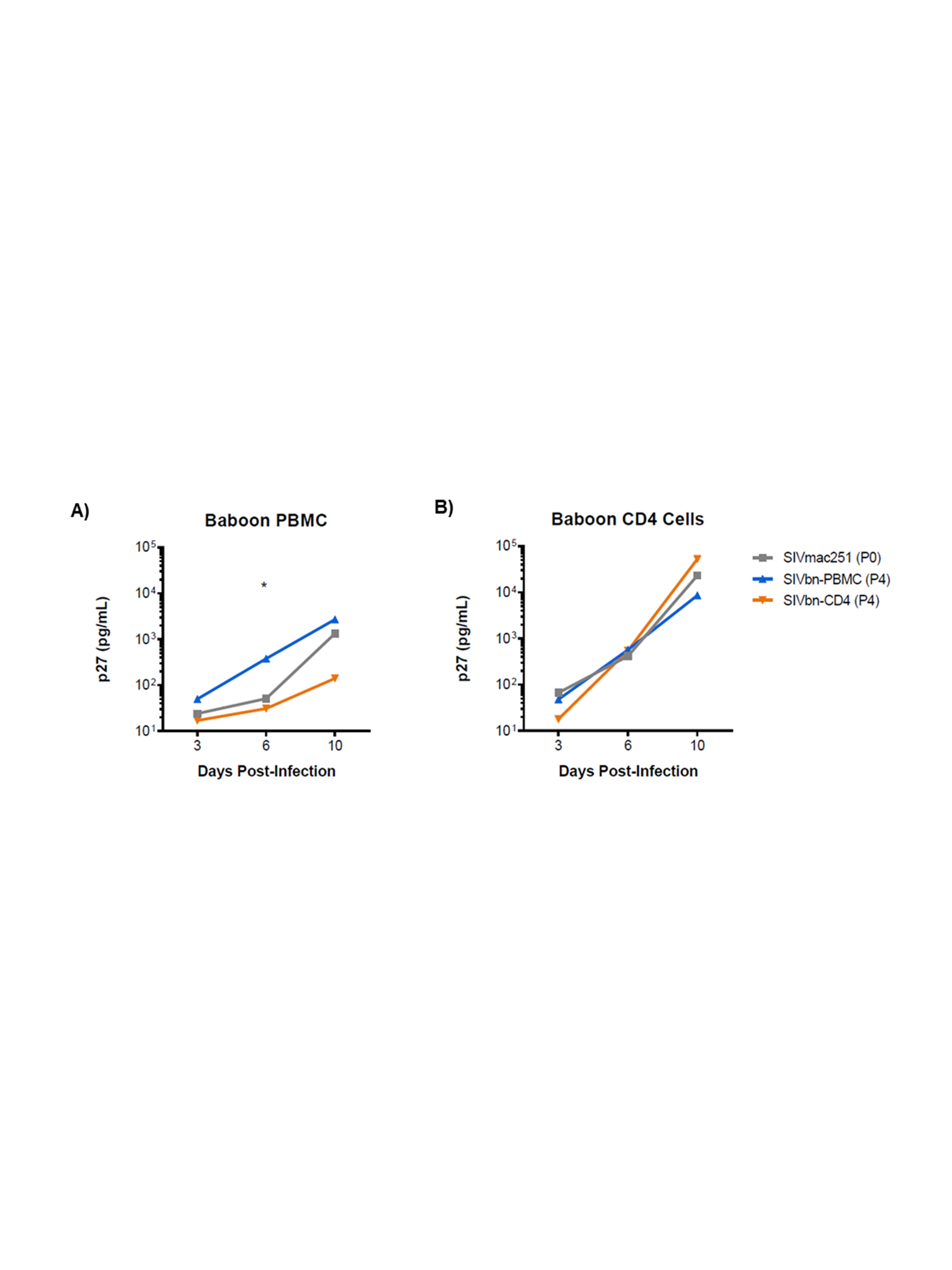

Supplement: Supplementary Figure 2 — SIVmac passaged in baboon PBMC, but not in CD4 cells, demonstrates adaptation for growth in Baboon PBMC. (A) PBMC and (B) CD4 cells were isolated from the same baboon donor and cultured for 48 hr in RPMI-10. Cells were infected with SIVmac251 (grey), SIVbn-PBMC P4S1 (blue), or SIVbn-CD4 (P4) (orange) at a M.O.I. of 100 g.e./cell. Viral concentrations were determined by measuring SIV p27 in the supernatant by Luminex. Data shown represents the median (n = 5). t-test was used for statistical analysis (*P ≤ 0.05). [file Image_2.tif]

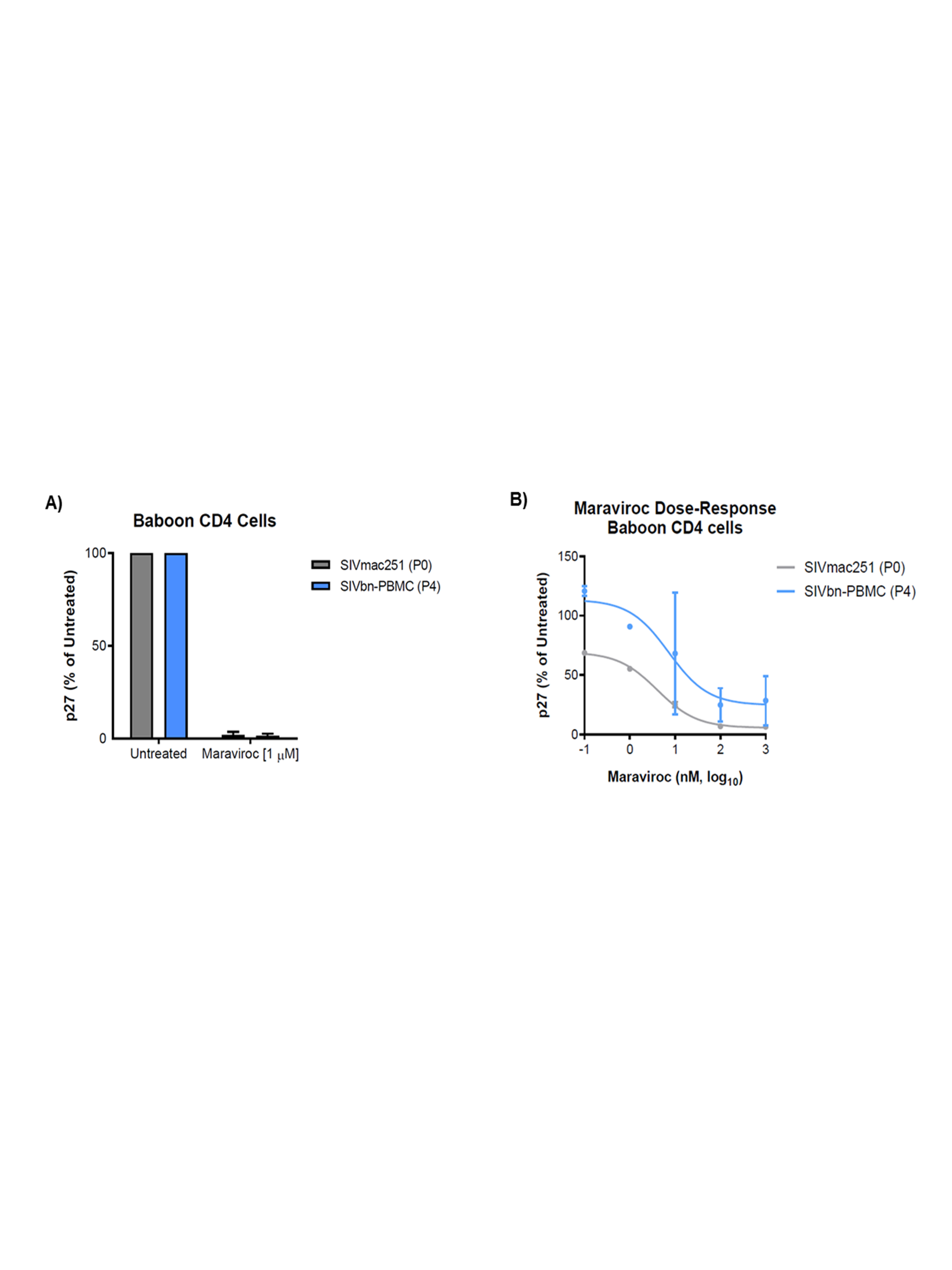

Supplement: Supplementary Figure 3 — SIVbn-PBMC P4 s1 has reduced sensitivity to CCR5 blockade. CD4 cells were isolated from baboon PBMC and cultured for 48 hr in RPMI-10. (A) CD4 cells were incubated with PBS (Untreated) or 1 µM maraviroc then infected with SIVmac251 or SIVbn-PBMC at a M.O.I of 0.01. PBS or maraviroc was supplemented in media during culture. Bar graph displays p27 values normalized to the untreated condition at 14 d.p.i. (mean ± SEM) (n = 2). (B) CD4 cells were infected with SIVmac251 or SIVbn-PBMC at a M.O.I of 0.01. Media was supplemented with PBS or one of 10-fold serial dilutions of maraviroc (0.1 nm – 1 µM). Dose-response curve is shown as linear regression on log-transformed maraviroc concentrations against p27 values normalized to untreated condition at 10 d.p.i. Bars indicate SEM (n = 2). All viral loads were determined by measuring p27 in the supernatant by Luminex. [file Image_3.tif]

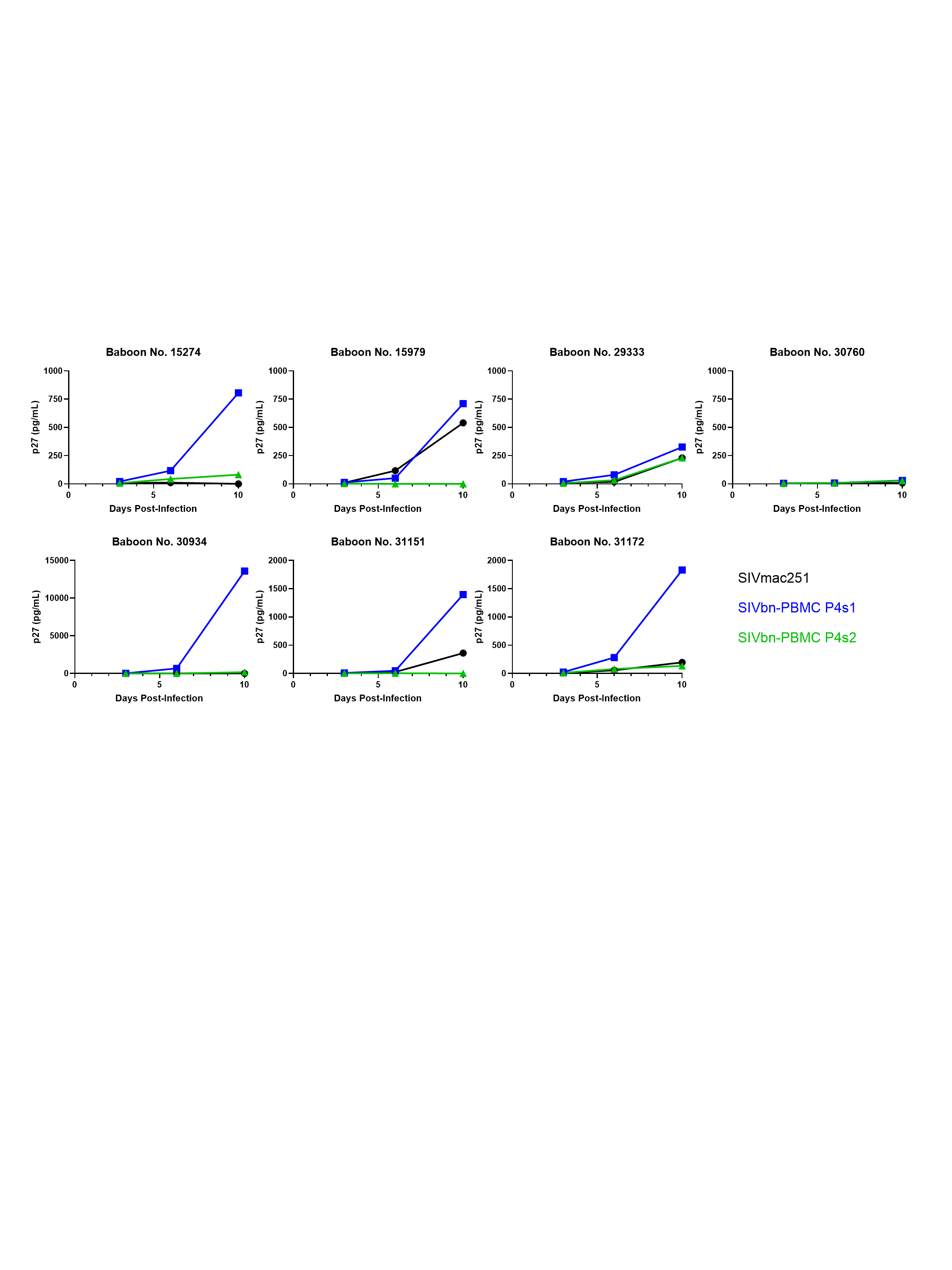

Supplement: Supplementary Figure 4 — SIVbn-PBMC P4 s1 has the highest replication efficiency in baboon PBMC. PBMC from seven naïve baboons were infected ex vivo with SIVmac251, or the baboon PBMC-adapted SIVbn-PBMC P4 s1 and PBMC P4 s2. SIVp27 concentration in the supernatant was quantified with a Luminex assay. [file Image_4.tif]
